# Dithiotreitol pre-treatment of synovial fluid samples improves microbiological counts in peri-prosthetic joint infection

**DOI:** 10.1007/s00264-023-05714-z

**Published:** 2023-02-22

**Authors:** Lorenzo Drago, Delia Romanò, Andrea Fidanza, Alessio Giannetti, Rocco Erasmo, Andreas F. Mavrogenis, Carlo Luca Romanò

**Affiliations:** 1 Clinical Microbiology, Department of Biomedical Sciences for Health, University of Milan, Milan, Italy; 2 Operative Unit of Osteoarticular Infection and Reconstructive Surgery, IRCCS Galeazzi S Ambrogio, Milan, Italy; 3 Department of Life, Health & Environmental Sciences-Mininvasive Orthopaedic Surgery, University of L’Aquila, L’Aquila, Italy; 4 Unit of Orthopaedics and Traumatology, Santo Spirito Hospital, Pescara, Italy; 5 First Department of Orthopaedics, School of Medicine, National and Kapodistrian University of Athens, Athens, Greece; 6 Studio Medico Associato Cecca-Romanò, Corso Venezia 2, 20121 Milan, Italy

**Keywords:** PJI, Synovial fluid, DTT, Microbiological culture examination, Biofilm

## Abstract

**Purpose:**

Synovial fluid cultures of periprosthetic joint infections (PJI) may be limited by bacteria living in the fluids as biofilm-aggregates. The antibiofilm pre-treatment of synovial fluids with dithiotreitol (DTT) could improve bacterial counts and microbiological early stage diagnosis in patients with suspected PJI.

**Methods:**

Synovial fluids collected from 57 subjects, affected by painful total hip or knee replacement, were divided into two aliquots, one pre-treated with DTT and one with normal saline. All samples were plated for microbial counts. Sensitivity of cultural examination and bacterial counts of pre-treated and control samples were then calculated and statistically compared.

**Results:**

Dithiothreitol pre-treatment led to a higher number of positive samples, compared to controls (27 vs 19), leading to a statistically significant increase in the sensitivity of the microbiological count examination from 54.3 to 77.1% and in colony-forming units count from 1884 ± 2.129 CFU/mL with saline pre-treatment to 20.442 ± 19.270 with DTT pre-treatment (*P* = 0.02).

**Conclusions:**

To our knowledge, this is the first report showing the ability of a chemical antibiofilm pre-treatment to increase the sensitivity of microbiological examination in the synovial fluid of patients with peri-prosthetic joint infection. If confirmed by larger studies, this finding may have a significant impact on routine microbiological procedures applied to synovial fluids and brings further support to the key role of bacteria living in biofilm-formed aggregates in joint infections.

## Introduction

Prosthetic joint infections (PJIs) represent one of the worst complications following joint arthroplasty, occurring at a rate of about 0.3 – 1.9% depending on the affected joint [1, 2], with a reported five year mortality rate that equals that of some of the most common oncological conditions [3].

Accurate and prompt diagnosis of PJI appears hence critical for a proper therapeutical approach, which is substantially different for septic and aseptic conditions.

In virtually all proposed diagnostic work-ups, microbiological investigations play a key role, with synovial fluid culture considered of paramount importance, allowing to identify the pathogen and its antibiotic susceptibility pattern before surgery through joint aspiration [4–6].

However, in spite of its pivotal role, current microbiological analysis of synovial fluid shows a remarkably low sensitivity, which may range from 41.6 to 90% [7–9].

Recently, the presence of bacteria and biofilm aggregates floating in the synovial fluids has been reported and associated to the recalcitrance of bacterial joint infections to common treatments [10–13].

Moreover, the metabolism of the microorganisms living as aggregates in synovial fluids has been shown to differ from that of bacteria living in planktonic state, a finding that may further complicate both the diagnostic and the therapeutical approaches to infections characterized by bacteria-colonizing fluids [14].

D-L dithiothreitol (DTT) is a sulfhydryl compound able to reduce disulfide bounds between polysaccharides and neighbor proteins, thus acting as an antibiofilm agent, without any toxicity on the living bacteria, if used at the right concentrations and time [15]. As such, DTT has been shown to increase the sensitivity of microbiological cultures both when applied to retrieved joint prosthetic implants and to the periprosthetic tissues [16–19], and even in cardiac valves [20].

We here hypothesized that the ability of bacteria to live in biofilm-based aggregates in the synovial fluid may be a reason for the relatively low sensitivity of traditional microbiological examination. Aim of the present study was hence to verify the hypothesis that the antibiofilm pre-treatment of synovial fluids with DTT is able to increase the sensitivity of cultural examination, by freeing the bacteria from the biofilm aggregates. To this purpose, we compared the rate of positive samples and the total amount of colonies grown after pre-treatment of synovial fluids with DTT or normal saline in patients affected by PJI or aseptic prosthetic loosening.

## Material and methods

Synovial fluid samples were collected by joint aspiration from 57 patients (35 females, 22 males, mean age 72.5 years, range: 57–82) undergoing hip or knee revision surgery in three orthopaedic centres.

The patients were classified as infected or not-infected according to the criteria established by the International Consensus Meeting of Philadelphia [5].

Residual (after routine analysis) synovial fluids were aseptically collected into 10-mL sterile tubes without any additive. The collection of synovial fluid has been performed by percutaneous joint aspiration using aseptic technique and with ultrasound guidance when necessary, particularly for aspiration of the hip joint. Each collected synovial fluid was divided into two equal aliquots (saline or DTT pre-treatment series) and inoculated onto appropriate culture media for aerobic and anaerobic microorganisms according to the AMCLI (Associazione Microbiologi Clinici Italiani) and the World Association against Infection in Orthopaedics and Trauma (WAIOT) procedures [21].

Those collected amounts of synovial fluid not sufficient for analysis were discarded.

Microbiological counts were then performed after the addition of saline or DTT pre-treatment.

In particular, samples were previously centrifuged at 3000 g for ten min, the supernatant discharged, and 1 ml of DTT (0.1%) or sterile saline was added to the pellet thus obtained by the split sample. All the samples were agitated for ten min at room temperature by an orbital shaker.

DTT 0.1% or saline pre-treated samples (0.1 ml) were inoculated in blood and chocolate agar plates, and incubated in aerobic and 10% CO2-enriched atmosphere, respectively, for three to five days at 36 ± 1 °C. For the anaerobic counts, samples were inoculated in Schaedler agar and incubated anaerobically for five to seven days at 36 ± 1 °C.

To compare the two pre-treatments (DTT vs saline), colonies grown on agar plates were counted. Microbial identification was performed on automated instrumentation Vitek 2 compact (BioMérieux, France).

### Statistical analysis

McNemar test on paired proportions (https://www.medcalc.org/calc/mcnemar.php) was used to compare the results of DTT and saline pre-treatments sensitivity. Colonies count were compared by means of paired Student’s *t* test (https://www.graphpad.com/quickcalcs/ttest1.cfm). Differences were considered statistically significant when the *P* value was less or equal to 0.05.

## Results

Thirty-five patients were defined as infected and 22 as not-infected, according to the MSIS definition.

Considering the agar plate counts, pre-treatment with DTT allowed to identify the pathogen in 27 patients, compared to 19 in the controls. Consequently, the overall sensitivity of microbiological examination increased from 54.3 to 77.1% in DTT pre-treated samples compared to normal saline (*P* < 0.0001), while the specificity did not show any difference (cf. Table 1).

**Table 1:** Comparison of microbiological count results with and without DTT pretreatment of samples

	Saline Pretreatment	DTT Pretreatment
True Positive	19	27
False Positive	0	0
False Negative	16	8
True Negative	22	22
Sensitivity	54.29% (36.65% to 71.17%)	77.14%(59.86% to 89.58%)
Specificity	100% (84.56% to 100.00%)	100% (84.56% to 100.00%)
Positive Predictive Value	100%	100%
Negative Predictive Value	99.08% (98.68% to 99.35%)	99.54% (99.15% to 99.75%)
Accuracy	99.09% (92.03% to 100.00%)	99.54% (92.85% to 100.00%)

Details of cultural count examination from samples pre-treated with saline or DTT in patients defined as infected are reported in Table 2.

**Table 2:** Comparison of microbiological count results with normal saline or DTT pretreatment of synovial fluid samples: isolated pathogens and colony forming units/mL count

Patient	Saline Pretreatment		Dithiothreitol Pretreatment	
	CFU/mL	Isolated microorganism(s)	CFU/mL	Isolated microorganism(s)
1	100	*Streptococcus sanguinis*	90	*Streptococcus sanguinis*
32	100	*Streptococcus sanguinis*	90	*Streptococcus sanguinis*
14	250	*Staphylococcus aureus*	3500	*Staphylococcus aureus*
3	720	*Escherichia coli, Staphylococcus aureus*	6500	*Escherichia coli, Staphylococcus aureus*
34	720	*Staphylococcus intermedius*	6500	*Staphylococcus intermedius*
2	980	*Enterococcus faecalis*	950	*Enterococcus faecalis*
33	980	*Enterococcus faecalis*	950	*Enterococcus faecalis*
17	1000	*Staphylococcus aureus*	32,000	*Staphylococcus aureus*
16	1500	*Escherichia coli*	25,000	*Escherichia coli*
28	2100	*Staphylococcus epidermidis*	2200	*Staphylococcus epidermidis*
15	2600	*Enterococcus faecalis*	12,600	*Enterococcus faecalis*
29	3900	*Streptococcus spp*	4300	*Streptococcus spp*
10	4400	*Enterococcus faecalis*	4200	*Enterococcus faecalis*
11	4800	*Staphylococcus epidermidis*	6400	*Staphylococcus epidermidis*
5	6400	*Staphylococcus aureus*	57,000	*Staphylococcus aureus*
18	71,000	*Staphylococcus lugdunensis*	71,000	*Staphylococcus lugdunensis*
27	92,000	*Staphylococcus aureus*	90,000	*Staphylococcus aureus*
23	100,000	*Staphylococcus aureus*	100,000	*Staphylococcus aureus*
24	100,000	*Staphylococcus aureus*	100,000	*Staphylococcus aureus*
13	Negative	-	1000	*Staphylococcus epidermidis*
22	Negative	-	1200	*Staphylococcus epidermidis*
30	Negative	-	1500	*Staphylococcus epidermidis*
7	Negative	-	2000	*Staphylococcus aureus*
8	Negative	-	3000	*Proteus mirabilis*
12	Negative	-	4200	*Streptococcus agalactiae*
21	Negative	-	4300	*Streptococcus agalactiae*
20	Negative	-	6500	*Staphylococcus aureus*
4	Negative	*-*	Negative	*-*
6	Negative	*-*	Negative	*-*
9	Negative	-	Negative	-
19	Negative	-	Negative	*-*
25	Negative	*-*	Negative	*-*
26	Negative	*-*	Negative	*-*
31	Negative	*-*	Negative	*-*
35	Negative	*-*	Negative	*-*

Similar bacterial counts with or without DTT pre-treatment were observed in 12 patients, while in the remaining seven, pre-treatment with dithiothreitol did show five- to 32-fold higher bacterial counts, with an average CFU/mL of 1.884 ± 2.129 with saline, compared to 20.442 ± 19.270 with DTT (*P* = 0.02).

In the eight cases in which no bacterial growth was observed with saline, but that resulted positive after DTT pre-treatment, the average bacterial count ranged from 1.000 to 6.500 CFU/mL (mean: 2.962 ± 1.922).

Notably, positive samples or higher bacterial counts were found not only when the pathogen was *Staphylococcus aureus*, which is known to live in aggregate form in fluids, but also for other *Staphylococci*, *Proteus mirabilis*, *E. coli*, and *E. faecalis*.

When both treatments were concordant, no differences were observed for isolated microorganisms, and no differences between treatments were found in time needed to yield positive cultures.

## Discussion

The major finding of this study is that dithiothreitol pre-treatment of synovial fluid samples, retrieved from patients with peri-prosthetic joint infection, allows to improve pathogen count and cultural examination sensitivity, if compared with control pre-treatment with normal saline. Moreover, in approximately one third of the cases in which the pathogen could be correctly identified even without the DTT pre-treatment, bacterial counts were significantly higher in the DTT pre-treated samples. Furthermore, the fact that an increase in bacterial count could be also observed for microorganisms other than *Staphylococci*, like *E. coli* and *Enterococcus faecalis*, suggests that the ability of bacteria to live in aggregates in the synovial fluids may be a common behavior among several if not all bacteria.

Overall, these original findings support the hypothesis that biofilm-bacteria aggregates in synovial fluids may play a role in the relatively low yield of current microbiological investigations and that antibiofilm chemical agents, like DTT, may be helpful to disrupt the biofilms, thus increasing the bacterial identification and count.

Analysis of synovial fluids represents an important tool for diagnosis of bone and joint infections, including prosthetic ones, since it can contribute to optimize patient management before surgery. In the recent past, it has been widely demonstrated that measurement of inflammatory markers in synovial fluids is a more specific approach than their evaluation in blood [22–24]. Nonetheless, culture remains crucial for the diagnosis of osteo-articular infections and particularly of PJI [25]. However, the usefulness of synovial fluid analysis is limited by the low sensitivity of cultures.

For this reason, in the recent years, several attempts have been done to improve microbiological analysis of synovial fluids by inoculating them into bottles for blood cultures or prolonging incubation time [9, 26]. Despite these efforts, synovial fluid culture still shows low sensitivity and, in many cases, diagnosis may be done only at the operative stage with cultures of peri-prosthetic tissues and/or prosthetic implant and histology [27]. Recently, it has been shown that the synovial fluid favors the formation of dense, protein and fluctuating biofilm aggregates which reduce the ability of antibiotics to reach microbial cells, similarly to the biofilm produced from the bacteria when adhering on a surface [13, 28]. The bacterial-protein interactions in these aggregates move variations in the production of virulence factors, phenotype and, finally, a marked tolerance to antibiotics similar to that described for adherent biofilms. In methicillin-resistant or methicillin-susceptible *Staphylococcus aureus* joint infections, the formation of strong aggregates has been associated to activity of specific surface proteins, modulated by activity of the accessory gene regulator (Agr) system which modulates the phenol-soluble modulins (PSMs) expression [29]. Agr-regulated virulence factors, specifically PSMs, are downregulated in synovial fluid. This downregulation appears to have consequences on synovial fluid-induced bacterial aggregation and an overexpression of any type of PSM (PSMα, PSMβ, and delta toxin) [30]. These findings support the notion that suppression of Agr and the concomitant strong suppression of PSM production are critical for MRSA aggregation and may be a target for possible novel therapeutical approaches [31]. While bacterial aggregates and their biofilms can be the target of new treatments, they should also probably be considered in view of finding more efficient microbiological diagnostics. In particular, the presence of biofilm-bacterial aggregates may have a strong impact on pathogen identification and on the bacterial count performed with traditional cultural techniques, that were designed for planktonic, isolated microorganisms. As DTT has been demonstrated to be able to remove microorganisms from the biofilms produced on prosthetic implants and on human tissues, we reasoned that it would eventually be effective in disrupting biofilm-bacteria aggregates in fluids and, more specifically, in the synovial fluid. It is evident that the destruction of the aggregated formation, at least in part facilitated by the binding of the protein components of the synovial fluid, likely can contribute to the identification of the bacterium as it increases the microbial recovery from this type of sampling.

The present study suffers of some limitations. The first is the limited number of patients. This is a limit common to many studies on PJI, due to the low incidence of these infections; moreover, the study required at least 2 mL of synovial fluid exceeding the sample volume routinely processed in the diagnostic workflow, but in some cases, the sample volume collected was not enough, thus reducing our population. This limitation prompts for larger trials to be performed in order to confirm our findings. A second limitation of the present research is the unique clinical condition investigated, which prevents to generalize the data to other conditions, as for example native joint infection. A further limit is the exclusion of joints other than knee and hip and the exclusion of patients that received antibiotic treatments at least 2 weeks prior to fluid samples collection. Moreover, it is worth considering that this investigation has been performed without broth enrichment and prolonged bacterial cultures, in order to test the ability of DTT to dissolve biofilm-bacteria aggregates through colony-forming units count. While this procedure did allow to demonstrate an increase of cultural examination sensitivity and a more accurate early detection of bacteria in the tested specimens, it does not add any information regarding the efficacy of DTT pre-treatment for prolonged cultures and after broth enrichment. This is in fact part of an ongoing further investigation.

These limitations notwithstanding, this study shows for the first time that the chemical antibiofilm pre-treatment with dithiothreitol is able to increase the sensitivity of cultural examination of synovial fluids in knee and hip PJIs. Furthermore, our findings support previous observations regarding the ability of bacteria to persist in biofilm-aggregates in synovial fluids, and bring new evidence that this can be a mode of living shared by various bacterial species, including Gram-negative microorganisms.
